# The impact of smoking cessation attempts on stress levels

**DOI:** 10.1186/s12889-019-6592-9

**Published:** 2019-03-06

**Authors:** Seong-Jun Kim, Wonjeong Chae, Woo-Hyun Park, Min-Ho Park, Eun-Cheol Park, Sung-In Jang

**Affiliations:** 1 Medical Courses, Yonsei University College of Medicine, Seoul, Republic of Korea; 2 Department of Public Health, College of Medicine, Yonsei University, Seoul, Republic of Korea; 3 Institute of Health Services Research, Yonsei University, Seoul, Republic of Korea; 4 Department of Preventive Medicine and Institute of Health Services Research, Yonsei University College of Medicine, 50 Yonsei-ro, Seodaemun-gu, Seoul, 120-752 Republic of Korea

**Keywords:** Smoking cessation attempt, Stress, Smoking cessation failure, Health policy

## Abstract

**Background:**

Cigarette smoking is a major health risk, particularly in male South Koreans. Smoking cessation can benefit health; however, the process of quitting smoking is difficult to some smokers and shows its relationship to their stress level. The hypothesis of this study is that who has failed attempts to stop smoking induce more stress than habitual smoking.

**Methods:**

To test this, the analysis on the association between smoking cessation attempts and stress levels in smokers was performed. The Korean Community Health Survey (2011–2016) data with the total of 488,417 participants’ data were used for this study. Survey data were analyzed using the chi-square test and logistic regression. As the dependent variable, self-reported level of stress was selected.

**Results:**

Of the subject population, 78.3% (63.3% males, 81.4% females) felt stressed. Among participants who successfully stopped smoking, 73.0% (72.6% males, 78.1% females) reported feeling stressed. In contrast, of those who failed to stop smoking, 83.3% (83.6% males, 86.3% females) reported high stress levels. Among those who did not attempt smoking cessation, 81.1% (81.2% males, 80.3% females) responded that they experienced stress. Those who failed to stop smoking had higher odds of stress than those who did not attempt smoking cessation [odds ratio (OR) 1.11, 95% confidence interval (CI) 1.09–1.14, *p* < 0.001]. Those who successfully stopped smoking had lower odds of stress than those who did not attempt smoking cessation (OR 0.87, 95% CI 0.86–0.89, p < 0.001).

**Conclusion:**

The study found an association between unsuccessful smoking cessation and stress level. As the result, people who failed smoking cessation showed higher stress. These data should be considered in health policy recommendations for smokers.

**Electronic supplementary material:**

The online version of this article (10.1186/s12889-019-6592-9) contains supplementary material, which is available to authorized users.

## Background

Cigarette smoking is a well-characterized underlying cause of cardiovascular and respiratory diseases, neoplasias, and depression [1–3]. Cessation of smoking is therefore recommended and is associated with many advantages. The risk of both smoking-related cancer, such as lung cancer, and other cancers was reduced by about 18–45% in one study that followed the health outcomes of former smokers [4]. Smoking cessation also reduced symptoms of depression and anxiety, while improving the overall quality of life for former smokers [5, 6].

In South Korea, 18.4% of adults smoked daily in 2015, which was slightly lower than the Organization for Economic Co-operation and Development (OECD) average of 18.5% [7]. Smoking rates were 40.7% among males and 6.4% among females in 2016. The male smoking rates have steadily declined from 66.3% in 1998 to 40.7% in 2016. In females, the smoking rates from 1998 to 2016 seemed consistent around 6.4% even though there was a point when it declined to 5.7% in 2014 [8]. The percentage of people who successfully quit smoking was 45.5%, with people who were older, married, or having high incomes demonstrating a better success rate for smoking cessation [9, 10].

The investigations conducted for years regarding the association between socioeconomic factors and smoking [11, 12] show that the prevalence of smoking is higher in low socioeconomic status [12, 13]. The relationship between smoking cessation and socioeconomic group, due to the supportive environment and motivation, they have advantages to succeeding in cessation [14]. Previous study discovered that where there was a less supportive environment for those with low socioeconomic status, they may experience difficulty in quitting [15–17]. As per motivation, the motivation to quit smoking is essential in the process of smoking cessation [14, 18]. The study [19] reported that those who were in low socioeconomic status groups showed low commitment or awareness of smoking cessation [19]. For example, the Hiscock, Judge, and Bauld [20] study highlighted that disadvantages of low socioeconomic level led to low successful cessation. Also, previous study discussed the results of quitting smoking and its correlation with occupational status [21].

Many studies have indicated that stress levels are a barrier for smoking cessation [9, 22] and that people with lower levels of stress have a better chance at successful cessation [9]. Some studies have reported that stress levels are associated with nicotine dependency rather than the smoking frequency, such that people who get stressed cannot easily quit smoking [23, 24]. Because perceived stress compounded with smoking greatly increases cardiovascular and hypothalamic-pituitary-adrenal measures like heart rate and blood pressure, cessation of smoking is critical for good health, despite stress-induced challenges [1].

There was previous research that investigated the mental health between people who had relapsed after quitting smoking and those who had entirely abstained. People who had relapsed displayed starkly increased anxiety when compared to those who had abstained [25]. However, despite the well-known impact of smoking on stress levels, it is hard to find studies investigating the association between stress levels and smoking cessation failure.

Stress is strongly related to smoking cessation and smoking relapse [26–28]. Smoking cessation can reduce stress, but before successful smoking cessation, it can contribute to stress to patients who are unsuccessful at quitting smoking. Cohen and Licthtenstein investigated the changes in stress levels in accordance with the status of smoking. The study reported that people who did not succeed in smoking cessation maintained the higher level of stress [27]. Also, the research conducted by Parrott discussed the reduced stress level after succeeding in smoking cessation. At this point, there is no adverse effect caused by acute nicotine depletion [28].

This study hypothesized that the stress associated with the experience of attempted smoking cessation could be detrimental; therefore, analysis of the association between attempted smoking cessation and stress levels was conducted.

## Methods

This study has used data from the Korean Community Health Survey (CHS) between 2011 and 2016, which is a national survey conducted by the Korean Centers for Disease Control and Prevention. The survey aims to establish a community health care plan, assess the viability of the plan, and produce comparable community health statistics. As representative data of the Korean population, trained surveyors conducted the survey in the computer-assisted personal interviewing method. A total of 253 households was chosen after multistage, stratified, and random selection of the local Korean communities by the resident registration. Each local community included around 900 participants. CHS survey included 229,226 people in 2011; 228,921 people in 2012; 228,781 people in 2013; 228,712 people in 2014; 228,558 people in 2015; and 228,452 people in 2016. CHS data are the secondary data available for public research.

Of the 1,372,650 subjects in the dataset (males: 618,051; females: 754,599), our study has included 506,396 people who were smokers (males: 465,177; females: 41,219) from 2011 to 2016. Of those, 488,417 subjects were included in the data analysis (males: 448,976; females: 39,441) and 17,979 subjects were excluded because of missing variables.

The variable-of-interest, that is smoking cessation is formed by the combination of two questions “Are you a current smoker?” and “Have you ever tried to quit smoking more than 24 hours?”. These questions express the smoking cessation attempt and its outcome. The variable is categorized into three groups: succeed (past-smoker and succeeded in cessation), failed (current-smoker and failed in cessation), and did not attempt (current-smoker and didn’t attempt smoking cessation).

The dependent variable was the stress levels, i.e., the existence of self-reported stress. The CHS inquiry regarding stress levels was “How much stress do you feel in your daily life?” and the response was multiple choice-based with 4 answers which are “I feel very much”, “I feel a lot”, “I feel a little bit” and “I hardly feel it”. The response was transformed into a binary response (High: I feel very much, I feel a lot, I feel a little bit; Low: I hardly feel it).

The independent variables included age, gender, family income, family members, marital status, education level, job, alcohol use, self-reported health, underlying chronic disease, and survey year.

Age was used as a categorized variable (6 groups: less than 20 years of age; 20 to 30 years of age; 30 to 40 years of age; 40 to 50 years of age; 50 to 60 years of age; and over 60 years of age). Family income variable was categorized into four groups (low: under 1,000,000 South Korean won/month; low-intermediate: 1,000,000-3,000,000 South Korean won/month; upper-intermediate: 3,000,000-5,000,000 South Korean won/month; high: over 5,000,000 South Korean won/month). The marital status variable was divided into three groups (marriage with cohabitation; single; else: other types of marriage). The education level variable was also categorized into four groups (under graduation from elementary school; dropout or graduation from middle school; dropout or graduation from high school; dropout or graduation from university or more). The job variable was categorized into three groups (office worker; site worker; unemployed or homemaker). The alcohol use variable was categorized into two groups (Yes: have drunk alcohol in the recent year; No: have not drunk alcohol in the recent year). The self-reported health status was divided into two categories (good; bad). The underlying chronic disease variable was determined based on whether the subject had experienced hypertension, diabetes, dyslipidemia, and arthritis diagnosed by doctors or not.

The data analysis was performed using multi logistic regression and chi-square tests. The analysis was performed on the fully adjusted model. Data analysis was performed with all subjects and then stratified by sex. The additional analyses were conducted on further levels of stress: high, mid, and low by sex (Additional file 1). Also, the sensitivity analysis on different level of stress was performed (Additional file 2). Subgroup analysis was done using stratification such variables as family income, family members, age, and marital status. Results were considered significant if *p*-value <.05. SAS 9.2 (SAS Institute Inc., Cary, NC) was used for data analysis.

## Results

Table 1 displays stress levels by independent variables and sex. Of the eligible respondents, 78.3% (63.3% of males and 81.4% of females) respond that they felt stressed daily. Of those who succeed in smoking cessation, 73.0% (72.6% of males and 78.1% of females) respond that they have experienced stress, while 83.8% of people who have failed in smoking cessation, (83.6% of male and 86.3% of female) respond that they have experienced stress. Of people who have even not tried to stop smoking, 81.1% (81.2% of males and 80.3% of females) responds that they have experienced stress. People who have used alcohol in the past year and have had higher education levels report that they have experienced stress. (Table 1).

**Table 1:** Association of stress, demographics, and clinical characteristics compared by sex (unit: individual, %)

		Total					Male					Female				
		Total		Yes	No	*p*-value	Total		High	Low	*p*-value	Total		High	Low	*p*-value
		N	%	%	%		N	%	%	%		N	%	%	%	
Smoking cessation	Succeed	226,030	46.3	73.0	27.0	< 0.001	208,903	46.5	72.6	27.4	< 0.001	17,127	43.4	78.1	21.9	< 0.001
	Failed	175,039	35.8	83.8	16.2		161,413	36.0	83.6	16.4		13,626	34.6	86.3	13.7	
	Did not attempt	87,348	17.9	81.1	18.9		78,660	17.5	81.2	18.8		8688	22.0	80.3	19.8	
Age	~ 20	2269	0.5	80.9	19.1	< 0.001	1965	0.4	79.1	20.9	< 0.001	304	0.8	92.4	7.6	< 0.001
	20 ~ 30	35,201	7.2	86.8	13.2		30,728	6.8	85.9	14.1		4473	11.3	92.4	7.6	
	30~40	76,086	15.6	91.1	8.9		69,099	15.4	91.0	9.0		6987	17.7	91.9	8.1	
	40~50	102,336	21.0	88.5	11.5		95,655	21.3	88.5	11.5		6681	16.9	87.9	12.1	
	50~60	104,658	21.4	80.4	19.6		98,092	21.9	80.1	19.9		6566	16.7	84.2	15.8	
	60~	167,867	34.4	63.3	36.7		153,437	34.2	62.8	37.2		14,430	36.6	68.5	31.6	
Sex	Male	448,976	91.9	78.1	22.0	< 0.001										
	Female	39,441	8.1	81.4	18.6											
Family income	High	125,653	25.7	81.6	18.4	< 0.001	119,466	26.6	81.5	18.5	< 0.001	6187	15.7	83.0	17.1	< 0.001
	Upper-intermediate	119,326	24.4	83.9	16.2		111,949	24.9	83.8	16.3		7377	18.7	85.4	14.7	
	Low-intermediate	162,073	33.2	77.5	22.5		147,703	32.9	76.8	23.2		14,370	36.4	85.1	14.9	
	Low	81,365	16.7	66.8	33.2		69,858	15.6	65.7	34.3		11,507	29.2	73.5	26.5	
Family number	1	48,956	10.0	75.8	24.2	< 0.001	38,300	8.5	76.2	23.8	< 0.001	10,656	27.0	74.2	25.8	< 0.001
	2	174,336	35.7	70.0	30.0		162,257	36.1	69.1	30.9		12,079	30.6	82.5	17.5	
	3	103,766	21.3	82.2	17.8		96,017	21.4	82.0	18.0		7749	19.7	85.1	14.9	
	4 and more	161,359	33.0	85.6	14.4		152,402	33.9	85.6	14.4		8957	22.7	85.4	14.6	
Marital status	Cohabiting marriage	363,908	74.5	77.5	22.5	< 0.001	346,873	77.3	77.1	22.9	< 0.001	17,035	43.2	86.2	13.8	< 0.001
	Other types of marriage	55,057	11.3	73.9	26.1		38,742	8.6	74.3	25.7		16,315	41.4	72.9	27.1	
	Single	69,452	14.2	86.0	14.0		63,361	14.1	85.5	14.5		6091	15.4	91.0	9.0	
Education level	University or more	167,768	34.4	85.3	14.8	< 0.001	160,001	35.6	85.1	14.9	< 0.001	7767	19.7	89.0	11.0	< 0.001
	High school	167,016	34.2	81.3	18.7		153,622	34.2	80.7	19.4		13,394	34.0	88.8	11.2	
	Middle school	61,343	12.6	72.0	28.0		57,169	12.7	71.1	28.9		4174	10.6	84.3	15.7	
	Under Elementary school	92,290	18.9	64.5	35.5		78,184	17.4	63.6	36.4		14,106	35.8	69.5	30.6	
Job	Office worker	99,546	20.4	88.5	11.6	< 0.001	95,257	21.2	88.3	11.7	< 0.001	4289	10.9	91.4	8.6	< 0.001
	Site worker	272,149	55.7	79.2	20.8		257,838	57.4	78.8	21.2		14,311	36.3	85.9	14.1	
	Unemployed or homemaker	116,722	23.9	67.6	32.4		95,881	21.4	65.7	34.3		20,841	52.8	76.3	23.7	
Alcohol use	Yes	389,832	79.8	80.6	19.5	< 0.001	363,894	81.1	80.2	19.8	< 0.001	25,938	65.8	85.3	14.7	< 0.001
	No	98,585	20.2	69.5	30.5		85,082	19.0	68.8	31.2		13,503	34.2	73.9	26.1	
Self-reported health condition	Good	393,605	80.6	78.7	21.3	< 0.001	367,593	81.9	78.5	21.5	< 0.001	26,012	66.0	81.9	18.1	< 0.001
	Bad	94,812	19.4	76.7	23.3		81,383	18.1	76.1	23.9		13,429	34.1	80.4	19.6	
Underlying Chronic Disease	Yes	184,916	37.9	73.4	26.6	< 0.001	168,407	37.5	73.2	26.8	< 0.001	16,509	41.9	76.1	23.9	< 0.001
	No	303,501	62.1	81.3	18.7		280,569	62.5	81.0	19.0		22,932	58.1	85.3	14.7	
Survey year	2011	78,725	16.1	79.3	20.7	< 0.001	72,530	16.2	79.1	20.9	< 0.001	6195	15.7	82.0	18.0	0.211
	2012	79,762	16.3	79.3	20.7		73,486	16.4	79.1	20.9		6276	15.9	82.1	17.9	
	2013	81,503	16.7	78.5	21.6		74,908	16.7	78.3	21.7		6595	16.7	80.6	19.4	
	2014	82,859	17.0	78.1	21.9		76,278	17.0	77.8	22.2		6581	16.7	81.3	18.7	
	2015	82,275	16.9	77.8	22.2		75,486	16.8	77.5	22.5		6789	17.2	81.1	18.9	
	2016	83,293	17.1	77.1	22.9		76,288	17.0	76.7	23.3		7005	17.8	81.6	18.4	
Total		488,417	100.0	78.3	21.7		448,976	100.0	78.1	22.0		39,441	100.0	81.4	18.6	

Table 2 shows the adjusted stress odds ratio of independent variables by sex. People who have succeed in smoking cessation decrease odds of stress compared to those who have even not tried to stop smoking [odds ratio (OR) 0.87, 95% confidence interval (CI) 0.86–0.89, *p* < 0.001]. When stratified by sex, both males (OR 0.88, 95% CI 0.86–0.90, p < 0.001) and females (OR 0.80, 95% CI 0.75–0.86, p < 0.001) continue to show significance.

**Table 2:** Adjusted binary logistic regressions to examine the association between stress, demographics and clinical characteristics compare by sex

		Total				Male				Female			
		OR	95% CI		*p*-value	OR	95% CI		*p*-value	OR	95% CI		*p*-value
Smoking cessation	Succeed	0.87	0.86	0.89	< 0.001	0.88	0.86	0.90	< 0.001	0.80	0.75	0.86	< 0.001
	Failed	1.11	1.09	1.14	< 0.001	1.10	1.08	1.13	< 0.001	1.18	1.09	1.27	< 0.001
	Did not attempt	1.00				1.00				1.00			
Age	~ 20	1.00				1.00				1.00			
	20 ~ 30	1.37	1.23	1.53	< 0.001	1.42	1.26	1.59	< 0.001	0.99	0.64	1.55	0.975
	30~40	1.74	1.55	1.94	< 0.001	1.86	1.66	2.09	< 0.001	0.96	0.62	1.50	0.869
	40~50	1.27	1.13	1.41	< 0.001	1.37	1.22	1.54	< 0.001	0.58	0.37	0.90	0.015
	50~60	0.75	0.67	0.84	< 0.001	0.80	0.71	0.90	< 0.001	0.44	0.28	0.69	< 0.001
	60~	0.40	0.36	0.45	< 0.001	0.43	0.38	0.48	< 0.001	0.23	0.15	0.37	< 0.001
Family income	High	0.91	0.89	0.94	< 0.001	0.93	0.91	0.96	< 0.001	0.70	0.63	0.78	< 0.001
	Upper-intermediate	1.01	0.98	1.04	0.556	1.03	1.00	1.06	0.033	0.76	0.69	0.84	< 0.001
	Low-intermediate	1.02	1.00	1.05	0.041	1.03	1.01	1.06	0.007	0.92	0.85	1.00	0.041
	Low	1.00				1.00				1.00			
Family number	1	1.00				1.00				1.00			
	2	0.94	0.91	0.95	< 0.001	0.92	0.89	0.95	< 0.001	1.23	1.13	1.33	< 0.001
	3	1.18	1.14	1.22	< 0.001	1.17	1.13	1.22	< 0.001	1.38	1.26	1.52	< 0.001
	4 and more	1.25	1.20	1.29	< 0.001	1.24	1.19	1.29	< 0.001	1.42	1.29	1.57	< 0.001
Marital status	Cohabiting marriage	1.19	1.15	1.23	< 0.001	1.19	1.15	1.23	< 0.001	1.06	0.94	1.20	0.327
	Other types of marriage	1.14	1.10	1.19	< 0.001	1.21	1.16	1.26	< 0.001	0.79	0.70	0.90	< 0.001
	Single	1.00				1.00				1.00			
Education level	University or more	1.00				1.00				1.00			
	High school	1.00	0.98	1.02	0.8395	0.99	0.97	1.02	0.529	1.10	0.99	1.21	0.071
	Middle school	0.94	0.92	0.97	< 0.001	0.93	0.91	0.96	< 0.001	1.10	0.97	1.26	0.148
	Under Elementary school	0.79	0.77	0.81	< 0.001	0.80	0.78	0.82	< 0.001	0.72	0.64	0.82	< 0.001
Job	Office worker	1.90	1.85	1.96	< 0.001	1.89	1.84	1.95	< 0.001	1.69	1.49	1.92	< 0.001
	Site worker	1.39	1.37	1.42	< 0.001	1.38	1.36	1.41	< 0.001	1.48	1.39	1.58	< 0.001
	Unemployed or homemaker	1.00				1.00				1.00			
Alcohol use	Yes	1.15	1.13	1.17	< 0.001	1.15	1.13	1.17	< 0.001	1.17	1.10	1.24	< 0.001
	No	1.00				1.00				1.00			
Self-reported health condition	Good	1.00				1.00				1.00			
	Bad	1.84	1.81	1.88	< 0.001	1.81	1.78	1.85	< 0.001	2.12	1.99	2.26	< 0.001
Underlying Chronic Disease	Yes	1.07	1.05	1.08	< 0.001	1.06	1.04	1.08	< 0.001	1.17	1.10	1.25	< 0.001
	No	1.00				1.00				1.00			
Survey year	2011	1.00				1.00				1.00			
	2012	1.02	1.00	1.05	0.101	1.02	1.00	1.05	0.083	1.00	0.90	1.10	0.933
	2013	1.00	0.98	1.03	0.8472	1.01	0.98	1.04	0.493	0.91	0.83	1.01	0.062
	2014	0.94	0.92	0.97	< 0.001	0.95	0.92	0.97	< 0.001	0.86	0.78	0.95	0.002
	2015	0.97	0.94	0.99	0.0112	0.98	0.95	1.00	0.083	0.85	0.78	0.94	< 0.001
	2016	0.94	0.92	0.97	< 0.001	0.95	0.92	0.97	< 0.001	0.87	0.79	0.96	0.005

(Table 2) People who have failed in smoking cessation increase odds of stress compared to those who have even not tried to stop smoking (OR 1.11, 95% CI 1.09–1.14, *p* < 0.001). When they were stratified by sex, this significance remained for both males (OR 1.10, 95% CI 1.08–1.13, p < 0.001), and for females (OR 1.18, 95% CI 1.09–1.27, *p* < 0.001). Based on the adjusted variables’ results, people who have self-reported that their health are bad have higher odds for stress compared to those who have self-reported that their health are good (OR 1.84, 95% CI 1.81–1.88, *p* < 0.001). The highest family income group has significantly lower odds for stress compared to the lowest family income group (OR 0.91, 95% CI 0.89–0.94, p < 0.001). When comparing with the number of household members, the group with more than 4 family members shows the highest odds for high stress levels. The odds ratio for stress is 0.94 (95% CI 0.91–0.95, *p* < 0.001 for the group whose number of family members is 2), 1.18 (95% CI 1.14–1.22, p < 0.001 for the group whose number of family members is 3), 1.25 (95% CI 1.20–1.29, p < 0.001 for the group whose number of family members is 4), compared to the group whose family number is 1. Based on these results, people who have underlying chronic disease have higher odds for stress than people who have not self-reported chronic disease (OR 1.07, 95% CI 1.05–1.08, *p* < 0.001). People who are current drinkers also have higher odds for stress than people who are not (OR 1.15, 95% CI 1.13–1.17, *p* < 0.001). The result of adjusted analysis on the stress level among those people who have attempted smoking cessation shows that when men have failed the cessation, the odds ratio of stress level is at 1.79 (95% CI 1.72–1.86, *p* < 0.001) and it is at 1.66 (95% CI 1.52–1.81, *p* < 0.001) in women (Additional file 3).

**Fig. 1:**
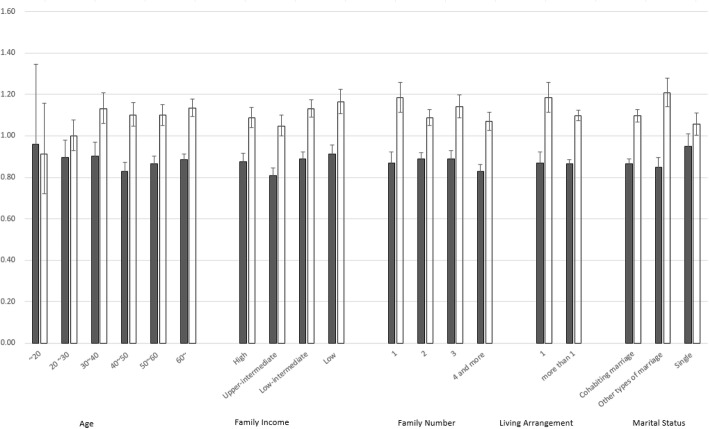
Stress odds for individuals who had attempted smoking cessation by subgroup for both sexes

Figure 1 displays the logistic regression results for associated stress in those who have attempted to quit smoking compared to those who have failed in smoking cessation after adjustment for all variables. When stratified by marital status, in the ‘else’ group, people who have failed in smoking cessation have significantly higher odds of stress than people who have even not attempted to stop smoking (for both sexes: OR 1.21, 95% CI 1.14–1.28, *p* < 0.001; males: OR 1.19, 95% CI 1.11–1.28, *p* < 0.001; females: OR 1.25, 95% CI 1.13–1.38, p < 0.001). (Fig. 1).

When stratified by the number of family members, the odds of stress in those who have failed in smoking cessation is significantly higher in the ‘living alone’ group (for both sexes: OR 1.18, 95% CI 1.19, p < 0.001; males: OR 1.19, 95% CI 1.11–1.27, p < 0.001; females: OR 1.20, 95% CI 1.05–1.36 *p* = 0.007), and the odds of stress for people who have succeed in smoking cessation is significantly lower in the ‘living alone’ group (for both sexes: OR 0.87, 95% CI 0.82–0.92, *p* < 0.001; for males: OR 0.91, 95% CI 0.85–0.97, *p* = 0.005; for females: OR 0.75, 95% CI 0.67–0.84, *p* < 0.001).

## Discussion

The study analyzed the association between smoking cessation experience and stress. The prevalence of stress in people who have failed to stop smoking was 1.11-fold higher than in those who have even not tried to stop smoking. Prevalence of stress in people who have successfully stopped smoking was 0.87-fold lower than in those who have even not tried to stop smoking. This association was stronger in females than in males. It might be one reason why the two genders had variable smoking cessation success rates. Variables such as income, family member number, and marital status were stratified for analyses. The result represented the trend that people who have failed to stop smoking had a higher risk of stress in most of the strata, especially among males. The results showed that smoking cessation reduced stress when they have succeeded. Smoking cessation can be harmful and elicit stress if the attempt fails.

There are many studies reporting results that are consistent with this study pertaining to the results about lower stress levels in the group that successfully quit smoking. Previous studies support the data that there is significant change in stress levels after and during smoking cessation. Pawalina et al. analyzed stress levels during smoking cessation treatment and found that the percentage of people who felt stress decreased from 62.68% before the start of program to 51.41% after the program [29]. There was a decrease in mean stress levels in the smoking cessation group (4.4 points, 95% CI 4.1–4.8) compared to the current-smoker group (5.2 points, 95% CI 4.9–5.6) in a study by Hajek et al. [30]. However, it was hard to find data about stress levels in the smoking-relapsed group in published reports.

Assessment of stress levels is important for those who have a smoking cessation plan. Incorporating a stress-coping skills program increase the success rates of smoking cessation (smoking cessation rate: 44% in the stress-coping skills program group; 27.5% in the control group) [31]. The stress is thought to be associated with nicotine dependency [23, 24], and nicotine dependency is related to higher rates of relapse and lower rates of smoking cessation [32]. Additionally, perceived stress determines smoking behavior [24]. For these reasons, increased stress levels for smoking cessation failure in our study have to be accounted for.

There are many factors that influence the perceived stress of smokers. Our results show that smoking cessation failure could impact the perceived stress of smokers. Skov-Ettrup et al. reported that people who had previously attempted to quit smoking got more stressed when they had stopped smoking [33]. People who have relapsed from attempted smoking cessation might therefore need more effort to quit smoking.

These stresses can impinge upon smoking addiction [34]. There are five stages of change during smoking cessation: pre-contemplation, contemplation, preparation, action, and maintenance [35]. Stress can impact the action and maintenance stages and result in relapse to cigarette smoking. This relationship between stress and smoking may be a result of the impact of stress on hypothalamic-pituitary-adrenal axis function and the autonomic nervous system [34].

Besides stress, many mental health problems are related to smoking [6]. Adults with serious psychological distress are likely to be smokers and to smoke heavily [36]. Smokers with any mental illness have lower self-reported quit rates and higher current- and lifetime- smoking rates compared to those without any mental illness [37]. However, depression is not associated with smoking cessation failure [38]. These mental health problems are usually rectified upon smoking cessation. Taylor et al. conducted a systematic review and reported the association between smoking cessation and mental health [5]. There were 4 studies about anxiety, 10 studies about depression, 5 studies about mixed anxiety and depression, 8 studies about the psychological quality of life, and 3 studies about stress after smoking cessation [5]. Regarding studied mental health elements, quitting smoking led to lowering the risk of mental health issues significantly [5]. Future studies will include these additional mental health parameters as variables for smoking cessation studies because no data on their association is currently available.

There are some limitations in this study. First, the study was based on the cross-sectional data that there was no causal relationship between smoking cessation and stress level and data that were collected at one point in time. Second, the period after the smoking cessation experience was not included in the analysis. Furthermore, data on the duration of smoking cessation attempts was lacking. Importantly, there were no standards measurement such as duration regarding smoking cessation. Therefore, the categorization of smoking cessation was placed into three categories for those attempting smoking cessation, and those completely succeeding in smoking cessation. Additionally, all data are computed based on self-reported variables and unconscious biases could be introduced. Thirdly, smoking cessation methods were not included in our analysis. Different cessation methods could lead to variable outcomes. Indeed, higher levels of behavioral counseling sessions resulted in higher rates of smoking cessation, but nicotine replacement therapy did not [39]. Individualized treatment also resulted in successful cessation [17].

## Conclusion

The study compares people who have succeeded in smoking cessation, have failed in smoking cessation, and have never attempted smoking cessation. The results show that people who have failed in quitting smoking experience more stress. Our study focuses on not only smoking cessation experience, but also the outcome of smoking cessation. Several reports have demonstrated that stress in smokers is associated with lower rates of smoking cessation and increased rates of cardiovascular and other diseases. Health policy and smoking cessation treatments should therefore be customized based on an individual’s past attempts to quit smoking to improve the success of cessation and associated health outcomes for former smokers.

## Additional files

Additional file 1: Appendix 1a Adjusted logistic regression to examine the association between stress levels (male). Appendix 1b Adjusted logistic regression to examine the association between stress levels (female). (DOCX 24 kb)

Additional file 2: Appendix 2 Binary logistic regression on stress: high level compare to mid-and low level. (DOCX 16 kb)

Additional file 3: Appendix 3 Comparison of the result of smoking cessation and stress level. (DOCX 15 kb)

## Abbreviations

CHS: Community Health Survey
OR: Odds ratio
