# Supplementary material for: The impact of smoking cessation attempts on stress levels
Source: BMC Public Health. 2019 Mar 6;19:267. doi: 10.1186/s12889-019-6592-9 (PMC6402150; doi:10.1186/s12889-019-6592-9)
Supplement: Supplementary file 1 — Appendix 1a Adjusted logistic regression to examine the association between stress levels (male). Appendix 1b Adjusted logistic regression to examine the association between stress levels (female). (DOCX 24 kb) [file 12889_2019_6592_MOESM1_ESM.docx]

Additional file 1a Adjusted logistic regression to examine the association between stress levels (male)

|  |  | Stress level high | | |  | Stress level medium | | |
| --- | --- | --- | --- | --- | --- | --- | --- | --- |
|  |  | OR | 95% CI | |  | OR | 95% CI | |
| Smoking Cessation | Succeed | 0.70 | 0.68 | 0.72 |  | 1.16 | 1.13 | 1.19 |
|  | Failed | 0.97 | 0.95 | 0.99 |  | 1.07 | 1.05 | 1.10 |
|  | Did not attempt | 1.00 |  |  |  | 1.00 |  |  |
| Age | ~20 | 1.00 |  |  |  | 1.00 |  |  |
|  | 20 ~30 | 1.38 | 1.21 | 1.58 |  | 1.44 | 1.28 | 1.62 |
|  | 30~40 | 1.87 | 1.63 | 2.14 |  | 1.84 | 1.63 | 2.08 |
|  | 40~50 | 1.21 | 1.06 | 1.39 |  | 1.45 | 1.28 | 1.63 |
|  | 50~60 | 0.58 | 0.50 | 0.66 |  | 0.92 | 0.82 | 1.04 |
|  | 60~ | 0.24 | 0.21 | 0.27 |  | 0.55 | 0.49 | 0.62 |
| Family income | High | 0.89 | 0.87 | 0.92 |  | 0.91 | 0.89 | 0.93 |
|  | Upper-intermediate | 1.00 | 0.97 | 1.02 |  | 1.00 | 0.98 | 1.03 |
|  | Low-intermediate | 1.09 | 1.06 | 1.12 |  | 0.93 | 0.90 | 0.95 |
|  | Low | 1.00 |  |  |  | 1.00 |  |  |
| Family number | 1 | 1.00 |  |  |  | 1.00 |  |  |
|  | 2 | 0.83 | 0.80 | 0.86 |  | 0.96 | 0.93 | 1.00 |
|  | 3 | 1.12 | 1.08 | 1.17 |  | 1.20 | 1.16 | 1.25 |
|  | 4 and more | 1.22 | 1.17 | 1.27 |  | 1.26 | 1.21 | 1.31 |
| Marital status | Cohabiting marriage | 1.39 | 1.34 | 1.45 |  | 1.10 | 1.06 | 1.14 |
|  | Other types of marriage | 1.55 | 1.47 | 1.62 |  | 1.07 | 1.03 | 1.12 |
|  | Single | 1.00 |  |  |  | 1.00 |  |  |
| Education level | University or more | 1.00 |  |  |  | 1.00 |  |  |
|  | High school | 0.96 | 0.94 | 0.99 |  | 1.01 | 0.99 | 1.03 |
|  | Middle school | 0.90 | 0.86 | 0.93 |  | 0.95 | 0.92 | 0.98 |
|  | Under Elementary school | 0.82 | 0.80 | 0.85 |  | 0.79 | 0.77 | 0.81 |
| Job | Office worker | 1.61 | 1.57 | 1.66 |  | 1.27 | 1.24 | 1.30 |
|  | Site worker | 0.73 | 0.71 | 0.75 |  | 0.72 | 0.70 | 0.73 |
|  | Unemployed or homemaker | 1.00 |  |  |  |  |  |  |
| Drinking status | Current drinker | 0.88 | 0.86 | 0.90 |  | 0.87 | 0.86 | 0.89 |
|  | Not-current drinker | 1.00 |  |  |  | 1.00 |  |  |
| Self-reported health condition | Good | 1.00 |  |  |  | 1.00 |  |  |
|  | Bad | 3.40 | 3.32 | 3.49 |  | 1.34 | 1.31 | 1.37 |
| Underlying Chronic Disease | Yes | 0.86 | 0.84 | 0.88 |  | 0.98 | 0.97 | 1.00 |
|  | No | 1.00 |  |  |  | 1.00 |  |  |
| Survey year | 2011 | 1.00 |  |  |  | 1.00 |  |  |
|  | 2012 | 1.01 | 0.98 | 1.04 |  | 1.03 | 1.00 | 1.06 |
|  | 2013 | 0.99 | 0.96 | 1.02 |  | 1.02 | 0.99 | 1.05 |
|  | 2014 | 0.95 | 0.92 | 0.98 |  | 0.95 | 0.92 | 0.97 |
|  | 2015 | 0.98 | 0.95 | 1.01 |  | 0.98 | 0.95 | 1.00 |
|  | 2016 | 0.95 | 0.92 | 0.98 |  | 0.95 | 0.92 | 0.98 |

b. Adjusted logistic regression to examine the association between stress levels (female)

|  |  | High level stress | | |  | Mid-level stress | | |
| --- | --- | --- | --- | --- | --- | --- | --- | --- |
|  |  | OR | 95% CI | |  | OR | 95% CI | |
| Smoking Cessation | Succeed | 0.69 | 0.64 | 0.75 |  | 0.90 | 0.84 | 0.97 |
|  | Failed | 1.26 | 1.16 | 1.37 |  | 1.11 | 1.02 | 1.20 |
|  | Did not attempt | 1.00 |  |  |  | 1.00 |  |  |
| Age | ~20 | 1.00 |  |  |  | 1.00 |  |  |
|  | 20 ~30 | 0.87 | 0.55 | 1.38 |  | 1.18 | 0.74 | 1.90 |
|  | 30~40 | 0.76 | 0.48 | 1.20 |  | 1.29 | 0.80 | 2.07 |
|  | 40~50 | 0.38 | 0.24 | 0.60 |  | 0.89 | 0.55 | 1.42 |
|  | 50~60 | 0.26 | 0.16 | 0.41 |  | 0.73 | 0.45 | 1.17 |
|  | 60~ | 0.12 | 0.07 | 0.19 |  | 0.43 | 0.27 | 0.70 |
| Family income | High | 0.81 | 0.74 | 0.89 |  | 1.02 | 0.94 | 1.11 |
|  | Upper-intermediate | 0.63 | 0.56 | 0.70 |  | 0.89 | 0.80 | 0.99 |
|  | Low-intermediate | 0.56 | 0.50 | 0.63 |  | 0.83 | 0.74 | 0.93 |
|  | Low | 1.00 |  |  |  | 1.00 |  |  |
| Family number | 1 | 1.00 |  |  |  | 1.00 |  |  |
|  | 2 | 1.34 | 1.23 | 1.47 |  | 1.14 | 1.04 | 1.24 |
|  | 3 | 1.58 | 1.42 | 1.75 |  | 1.23 | 1.11 | 1.37 |
|  | 4 and more | 1.67 | 1.50 | 1.86 |  | 1.24 | 1.12 | 1.38 |
| Marital status | Cohabiting marriage | 1.09 | 0.96 | 1.24 |  | 1.05 | 0.93 | 1.20 |
|  | Other types of marriage | 0.77 | 0.67 | 0.88 |  | 0.82 | 0.72 | 0.93 |
|  | Single | 1.00 |  |  |  | 1.00 |  |  |
| Education level | University or more | 1.00 |  |  |  |  |  |  |
|  | High school | 1.11 | 1.00 | 1.23 |  | 1.08 | 0.98 | 1.20 |
|  | Middle school | 1.12 | 0.97 | 1.29 |  | 1.09 | 0.95 | 1.25 |
|  | Under Elementary school | 0.76 | 0.66 | 0.87 |  | 0.70 | 0.61 | 0.80 |
| Job | Office worker | 1.22 | 1.07 | 1.40 |  | 1.08 | 0.94 | 1.23 |
|  | Site worker | 0.59 | 0.55 | 0.63 |  | 0.75 | 0.70 | 0.81 |
|  | Unemployed or homemaker | 1.00 |  |  |  | 1.00 |  |  |
| Drinking status | Current drinker | 0.88 | 0.82 | 0.94 |  | 0.84 | 0.78 | 0.89 |
|  | Not-current drinker | 1.00 |  |  |  | 1.00 |  |  |
| Self-reported health condition | Good | 1.00 |  |  |  |  |  |  |
|  | Bad | 3.50 | 3.25 | 3.77 |  | 1.41 | 1.31 | 1.51 |
| Underlying Chronic Disease | Yes | 0.78 | 0.72 | 0.84 |  | 0.92 | 0.86 | 0.99 |
|  | No | 1.00 |  |  |  | 1.00 |  |  |
| Survey year | 2011 | 1.00 |  |  |  | 1.00 |  |  |
|  | 2012 | 0.99 | 0.89 | 1.10 |  | 1.01 | 0.91 | 1.11 |
|  | 2013 | 0.90 | 0.81 | 1.00 |  | 0.93 | 0.84 | 1.02 |
|  | 2014 | 0.85 | 0.76 | 0.94 |  | 0.87 | 0.79 | 0.96 |
|  | 2015 | 0.87 | 0.78 | 0.96 |  | 0.84 | 0.76 | 0.93 |
|  | 2016 | 0.87 | 0.78 | 0.96 |  | 0.88 | 0.79 | 0.97 |
