# Supplementary material for: The impact of smoking cessation attempts on stress levels
Source: BMC Public Health. 2019 Mar 6;19:267. doi: 10.1186/s12889-019-6592-9 (PMC6402150; doi:10.1186/s12889-019-6592-9)
Supplement: Supplementary file 2 — Appendix 2 Binary logistic regression on stress: high level compare to mid-and low level. (DOCX 16 kb) [file 12889_2019_6592_MOESM2_ESM.docx]

Additional file 2. Binary logistic regression on stress: high level compare to mid-and low level

|  |  | Male | | |  | Female | | |
| --- | --- | --- | --- | --- | --- | --- | --- | --- |
|  |  | OR | 95% CI | |  | OR | 95% CI | |
| Smoking Cessation | Succeed | 0.63 | 0.60 | 0.66 |  | 0.66 | 0.60 | 0.73 |
|  | Failed | 1.12 | 1.07 | 1.17 |  | 1.09 | 1.00 | 1.20 |
|  | Did not attempt | 1.00 |  |  |  | 1.00 |  |  |
| Age | ~20 | 1.00 |  |  |  | 1.00 |  |  |
|  | 20 ~30 | 1.01 | 0.81 | 1.24 |  | 0.80 | 0.58 | 1.11 |
|  | 30~40 | 1.03 | 0.83 | 1.28 |  | 0.60 | 0.43 | 0.84 |
|  | 40~50 | 0.73 | 0.59 | 0.91 |  | 0.41 | 0.29 | 0.57 |
|  | 50~60 | 0.47 | 0.38 | 0.58 |  | 0.33 | 0.23 | 0.47 |
|  | 60~ | 0.23 | 0.19 | 0.29 |  | 0.19 | 0.13 | 0.28 |
| Family income | High | 0.78 | 0.74 | 0.82 |  | 0.76 | 0.68 | 0.84 |
|  | Upper-intermediate | 0.75 | 0.70 | 0.80 |  | 0.65 | 0.57 | 0.75 |
|  | Low-intermediate | 0.77 | 0.72 | 0.82 |  | 0.73 | 0.64 | 0.85 |
|  | Low | 1.00 |  |  |  | 1.00 |  |  |
| Family number | 1 | 1.00 |  |  |  | 1.00 |  |  |
|  | 2 | 0.83 | 0.78 | 0.88 |  | 1.22 | 1.09 | 1.36 |
|  | 3 | 0.89 | 0.83 | 0.95 |  | 1.19 | 1.04 | 1.35 |
|  | 4 and more | 0.92 | 0.86 | 0.99 |  | 1.30 | 1.14 | 1.48 |
| Marital status | Cohabiting marriage | 1.31 | 1.24 | 1.39 |  | 1.06 | 0.93 | 1.20 |
|  | Other types of marriage | 1.58 | 1.47 | 1.70 |  | 0.91 | 0.79 | 1.04 |
|  | Single | 1.00 |  |  |  | 1.00 |  |  |
| Education level | University or more | 1.00 |  |  |  | 1.00 |  |  |
|  | High school | 0.92 | 0.89 | 0.96 |  | 0.97 | 0.87 | 1.08 |
|  | Middle school | 0.87 | 0.82 | 0.93 |  | 1.00 | 0.85 | 1.17 |
|  | Under Elementary school | 0.90 | 0.84 | 0.96 |  | 0.91 | 0.77 | 1.08 |
| Job | Office worker | 1.37 | 1.32 | 1.43 |  | 1.04 | 0.92 | 1.19 |
|  | Site worker | 1.19 | 1.13 | 1.25 |  | 0.83 | 0.77 | 0.91 |
|  | Unemployed or homemaker | 1.00 |  |  |  | 1.00 |  |  |
| Drinking status | Current drinker | 1.00 | 0.96 | 1.05 |  | 1.04 | 0.95 | 1.14 |
|  | Not-current drinker | 1.00 |  |  |  | 1.00 |  |  |
| Self-reported health condition | Good | 1.00 |  |  |  |  |  |  |
|  | Bad | 3.76 | 3.61 | 3.91 |  | 3.42 | 3.13 | 3.72 |
| Underlying Chronic Disease | Yes | 0.84 | 0.81 | 0.87 |  | 0.91 | 0.82 | 1.00 |
|  | No | 1.00 |  |  |  | 1.00 |  |  |
| Survey year | 2011 | 1.00 |  |  |  | 1.00 |  |  |
|  | 2012 | 1.07 | 1.01 | 1.13 |  | 1.14 | 1.00 | 1.30 |
|  | 2013 | 1.05 | 0.99 | 1.11 |  | 1.07 | 0.94 | 1.23 |
|  | 2014 | 1.26 | 1.20 | 1.33 |  | 1.24 | 1.09 | 1.41 |
|  | 2015 | 1.18 | 1.11 | 1.24 |  | 1.15 | 1.01 | 1.31 |
|  | 2016 | 1.24 | 1.18 | 1.31 |  | 1.28 | 1.13 | 1.46 |

†Multilevel logistic regress was conducted.
