# Supplementary material for: The impact of smoking cessation attempts on stress levels
Source: BMC Public Health. 2019 Mar 6;19:267. doi: 10.1186/s12889-019-6592-9 (PMC6402150; doi:10.1186/s12889-019-6592-9)
Supplement: Supplementary file 3 — Appendix 3 Comparison of the result of smoking cessation and stress level. (DOCX 15 kb) [file 12889_2019_6592_MOESM3_ESM.docx]

Additional file 3. Comparison of the result of smoking cessation and stress level

|  |  | Male | | | Female | | |
| --- | --- | --- | --- | --- | --- | --- | --- |
|  |  | OR | 95% CI | | OR | 95% CI | |
| **Smoking Cessation** | **Failed** | 1.79 | 1.72 | 1.86 | 1.66 | 1.52 | 1.81 |
|  | **Succeed** | 1.00 |  |  | 1.00 |  |  |
| Age | ~20 | 1.00 |  |  | 1.00 |  |  |
|  | 20 ~30 | 0.98 | 0.76 | 1.26 | 0.79 | 0.54 | 1.14 |
|  | 30~40 | 1.00 | 0.78 | 1.29 | 0.59 | 0.40 | 0.86 |
|  | 40~50 | 0.70 | 0.54 | 0.90 | 0.39 | 0.26 | 0.57 |
|  | 50~60 | 0.45 | 0.34 | 0.58 | 0.33 | 0.22 | 0.48 |
|  | 60~ | 0.22 | 0.17 | 0.29 | 0.18 | 0.12 | 0.28 |
| Family income | High | 0.78 | 0.73 | 0.83 | 0.78 | 0.69 | 0.89 |
|  | Upper-intermediate | 0.75 | 0.70 | 0.80 | 0.66 | 0.57 | 0.78 |
|  | Low-intermediate | 0.78 | 0.72 | 0.83 | 0.75 | 0.63 | 0.88 |
|  | Low | 1.00 |  |  | 1.00 |  |  |
| Family number | 1.00 | 1.00 |  |  |  |  |  |
|  | 2.00 | 0.83 | 0.78 | 0.90 | 1.23 | 1.08 | 1.41 |
|  | 3.00 | 0.91 | 0.84 | 0.98 | 1.20 | 1.04 | 1.39 |
|  | 4 and more | 0.92 | 0.85 | 1.00 | 1.34 | 1.15 | 1.55 |
| Marital status | Cohabiting marriage | 1.28 | 1.20 | 1.37 | 1.08 | 0.94 | 1.25 |
|  | Other types of marriage | 1.57 | 1.45 | 1.70 | 0.94 | 0.80 | 1.10 |
|  | Single | 1.00 |  |  | 1.00 |  |  |
| Education level | University or more | 1.00 |  |  | 1.00 |  |  |
|  | High school | 0.91 | 0.87 | 0.95 | 0.96 | 0.86 | 1.08 |
|  | Middle school | 0.85 | 0.79 | 0.92 | 1.03 | 0.86 | 1.23 |
|  | Under Elementary school | 0.91 | 0.84 | 0.98 | 0.96 | 0.79 | 1.17 |
| Job | Office worker | 1.35 | 1.29 | 1.41 | 1.05 | 0.91 | 1.21 |
|  | Site worker | 1.19 | 1.13 | 1.26 | 0.84 | 0.76 | 0.92 |
|  | Unemployed or homemaker | 1.00 |  |  | 1.00 |  |  |
| Drinking status | Current drinker | 1.01 | 0.96 | 1.07 | 1.03 | 0.93 | 1.14 |
|  | Not-current drinker | 1.00 |  |  | 1.00 |  |  |
| Self-reported health condition | Good | 1.00 |  |  | 1.00 |  |  |
|  | Bad | 3.68 | 3.52 | 3.85 | 3.45 | 3.13 | 3.81 |
| Underlying Chronic Disease | Yes | 0.84 | 0.80 | 0.87 | 0.94 | 0.84 | 1.05 |
|  | No | 1.00 |  |  | 1.00 |  |  |
| Survey year | 2011.00 | 1.00 |  |  | 1.00 |  |  |
|  | 2012.00 | 1.06 | 0.99 | 1.13 | 1.09 | 0.94 | 1.27 |
|  | 2013.00 | 1.02 | 0.96 | 1.09 | 1.05 | 0.90 | 1.22 |
|  | 2014.00 | 1.24 | 1.16 | 1.31 | 1.24 | 1.07 | 1.44 |
|  | 2015.00 | 1.14 | 1.07 | 1.21 | 1.10 | 0.95 | 1.28 |
|  | 2016.00 | 1.23 | 1.16 | 1.31 | 1.19 | 1.03 | 1.38 |
| † Excluded who did not attempt the smoking cessation | |  |  |  |  |  |  |
